# Trace and toxic element patterns in nonsmoker patients with noninsulin-dependent diabetes mellitus, impaired glucose tolerance, and fasting glucose

**DOI:** 10.4103/0973-3930.50713

**Published:** 2009

**Authors:** Muhittin A. Serdar, Fatih Bakir, Adnan Haşimi, Tuğrul Çelik, Okhan Akin, Levent Kenar, Osman Aykut, Metin Yildirimkaya

**Affiliations:** 1 Ankalab Laboratories, Ankara, Turkey; 2 Gulhane School of Medicine, Department of Clinical Chemistry, Turkey; 3 Numune Education and Research Hospital, Biochemistry Department, Turkey; 4 Kecioren Education and Research Hospital, Biochemistry Department, Turkey; 5 Refik Saydam Hygiene Center, Ankara 06100, Turkey

**Keywords:** Diabetes mellitus, inductively coupled plasma mass spectrometry, trace elements

## Abstract

**PROJECT:**

Noninsulin dependent diabetes mellitus is supposed to be associated with fluctuations in the plasma levels of several trace elements. There is accumulating evidence that the metabolism of several trace elements is altered in patients with noninsulin dependent diabetes mellitus and that these nutrients might have specific roles in the pathogenesis and progression of this disorder.

**PROCEDURE:**

The aim of the present study is to compare the levels of essential trace and toxic elements including lead (Pb), arsenic (As), cadmium (Cd), chromium (Cr), aluminium (Al), nickel (Ni), cobalt (Co), iron (Fe), copper (Cu), selenium (Se), zinc (Zn), vanadium (V), manganese (Mn), barium (Ba), silver (Ag), and mercury (Hg) in patients with noninsulin dependent diabetes mellitus (*n* = 31), impaired glucose tolerance (*n* = 20), impaired fasting glucose (*n* = 14), and healthy controls (*n* = 22). Plasma concentrations of the elements were measured by using inductively coupled plasma mass spectrometry.

**RESULTS:**

The results indicated that values of lead, nickel, aluminium, copper, and chromium were significantly higher, but not above toxic levels, in the plasma of nonsmoker patients with noninsulin dependent diabetes mellitus (*P* < 0.05). The values for these elements were found to be significantly higher (*P* < 0.05) in patients with impaired fasting glucose than in controls. Moreover, a statistically significant correlation was found between plasma levels of glycated hemoglobin and of some trace elements like lead, nickel, aluminium, copper, chromium, cadmium, and mercury.

**CONCLUSIONS:**

Thus, it was concluded that chronic complications of glucose metabolism disorders might be associated with alterations in the levels of some trace elements. Nevertheless, some more timely and extensive studies are required to clarify the exact mechanisms of each of these changes.

## Introduction

A number of studies have reported that the metabolism and levels of several trace elements might be altered in noninsulin-dependent diabetes mellitus (NIDDM) and that these elements have specific roles in either pathogenesis or progression of this disorder.[1,2]

This study aims to compare the plasma levels of trace elements including Pb, As, Cd, Cr, Al, Ni, V, Mn, Co, Zn, Cu, Se, Ba, Fe, and Hg in NIDDM patients, in patients with impaired glucose tolerance (IGT), and those with impaired fasting glucose (IFG).

## Material and Methods

The study population consisted of nonsmoker patients with NIDDM (*n* = 31), IGT (*n* = 20), and IFG (*n* = 14) as well as nonsmoker healthy controls (*n* = 22). Obese NIDDM patients who had been regularly attending the Diabetes Clinic as outdoor patients were recruited over six months for a clinical trial on the effects of antihypertensive treatment on the insulin sensitivity. The patients possessing the following criteria were included in the study: age 30–70 years; body mass index (BMI) < 30 kg/m2; no treatment with insulin or any other drugs known to influence the glucose metabolism; no history of any recent acute illness or clinical evidence suggestive of kidney, liver, or endocrine diseases; no severe chronic diabetic complications (proliferative retinopathy, albuminuria, symptomatic neuropathy, coronary, and other vascular diseases. Patients taking vitamin and/or mineral supplements were excluded from the study.

Plasma levels of Pb, As, Cd, Cr, Al, Ni, V Mn, Co, Zn, Cu, Se, Ag, Ba, Fe, and Hg were measured in the present study. Blood samples were drawn with metal-free, stainless steel needles into appropriately-coated tubes (Becton Dickinson Laboratories, Franklin Lakes, NJ, USA) which were centrifuged at 2000 × *g* for ten minutes. The samples were tested for trace elements by using Agilent 7500cx Inductively Coupled Plasma Mass Spectrometry (ICP-MS) (Agilent Technologies, USA). Instrumental operating conditions for ICP-MS are shown in Table 1. Glycated hemoglobin (HbA1c) levels were determined by using the ready-to-use reagent kit for HPLC analysis (Chromsystems; München, Germany). Glucose measurements were carried out by the photometric hexokinase method by using the Advia 1800 chemistry analyzer (Siemens Healthcare Diagnostics, IL, USA). The insulin C peptide levels were measured by using the Advia Centaur chemiluminescent analyzer (Siemens Healthcare Diagnostics, IL, USA).

**Table 1:** Instrumental operating conditions for ICP-MS

Spectrometer	Mass
Integration time	3 /point = 9/mass for Hg
RF power (W)	1500
Plasma gas flow rate (L/min)	(Argon) 15
Auxiliary gas flow rate (L/min)	0.90
Carrier gas flow rate (L/min)	1.06
Sampling depth (mm)	7.1
Acquisition Mode	Spectrum
Number Replicates	3
Cone	Nickel

Impaired glucose tolerance (IGT), a kind of prediabetic state of dysglycemia, may be defined as two-hour glucose levels of 140–199 mg/dL (7.8–11.0 mmol/L) on the 75-g oral glucose tolerance test. Impaired fasting glucose (IFG) is defined as fasting glucose levels of 100–125 mg/dL (5.6–6.9 mmol/L). These glucose levels are above normal levels but below the level that is diagnostic for diabetes mellitus. From this point of view, patients with IGT or IFG who are supposed to have significant risk of progressing to diabetes, represent an essential target group for primary protection against more severe pathologies.

The resulting data were analyzed by using SPSS for Windows software. All results were analyzed by applying the one-sample Kolmogorov-Smirnov test to determine the normal distribution. The Mann-Whitney U-test was used to determine the differences between the groups. The correlations were found using the Spearman correlation; *P* < 0.05 was considered to be statistically significant.

## Results

**Figure 1:**
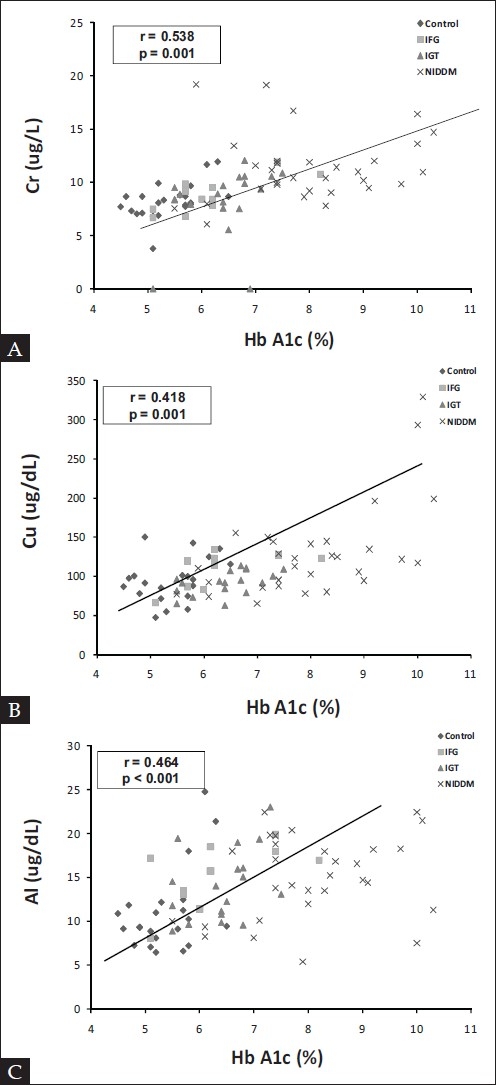
Correlation between HbA1c and trace element levels (A) Cr, (B) Cu, (C) Al

Levels of Hg, Ni, Al, and Cr in plasma samples of NIDDM patients were significantly higher (but well below the toxic levels for all of them) than in the controls (*P* < 0.05) [Table 2]. There was a statistically significant correlation among plasma glucose, HbA1c and Cu, Al, V, Ni, Cd, and Pb [Table 3]. Furthermore, significantly higher levels were noted for only Al and V in the plasma of patients with IFG and IGT as compared to controls. For the IGT and IFG patients, despite a slight increase in the mean plasma concentrations of Fe, Cr, Cu, and Pb, no statistically significant difference was demonstrated in comparison with the control group [Figure 1].

**Table 2:** HbA1c and trace element levels of the groups

	Reference values	Control *n*=22	IFG *n*=14	IGT *n*=20	NIDDM *n*=31
Age (years)		52 ± 13	55 ± 10	58 ± 11	59 ± 9
Hb A1c (%)	< 6	5.38 ± 0.55	6.25 ± 1.13*	6.31 ± 0.82*	7.98 ±1.29*
HOMA index		1.18 ± 0.59	2.17 ± 0.92*	1.66 ± 0.74	1.97 ± 1.22*
Insulin (U/L)	< 25	8.18 (5.64–11.5)	11.4 (10.1–22.4)*	11.65 (10.04–12.4)	10.2 (7.17–13.7)
Aluminium (μg/L)	< 15	11.4 ± 4.7	14.7 ± 3.26*	14.1 ± 4.07*	15.4 ± 5.21*
Iron (μg/dL)	65–170	93.2 ± 43.2	101 ± 48.7	95.6 ± 38.2	99.9 ± 64.5
Nickel (μg/L)	< 5.2	0.52 ± 0.25	0.65 ± 0.29	0.79 ± 0.66	1.01± 0.62*
Cobalt (μg/L)	0.1–0.4	0.52 ± 0.37	0.50 ± 0.38	0.50 ± 0.37	0.53 ± 0.39
Copper (μg/dL)	70–155	94.6 ± 28.2	102 ± 21.6	92.0 ± 15.1	122 ± 32.9*
Silver (μg/L)	< 1	ND	ND	ND	0(0–0.1)
Arsenic (μg/L)	< 15.4	0.86 (0.64–1.59)	1.65 ± 0.87	1.59 ± 0.97	1.22 ± 0.57
Selenium (μg/L)	23–190	123 ± 42.9	123 ± 21.2	120 ± 29.2	127 ± 36.1
Cadmium (μg/L)	< 0.2	0.01 (0.01–0.02)	0.01 (0.01–0.10)	0.01 (0.01–0.06)	0.01 (0.01–0.08)
Barium (μg/L)	< 10	5.91 ± 2.79	6.02 ± 3.5	5.17± 3.45	5.75 ± 3.99
Mercury (μg/L)	< 5.8	1.53 ± 0.69	1.08 ± 0.23	1.76 (1.04–1.91)	1.15 (1.02–1.75)
Lead (μg/dL)	< 24.9	0.01 (0.01–0.02)	0.01 (0.01–0.307)	0.01 (0.01–0.327)	0 (0–1.11)*
Zinc (μg/dL)	65–291	81.9 ± 29.6	84.2 ±18.8	78.3 ± 19.8	88.4 ± 56.4
Vanadium (μg/L)	< 1	0.45 (0.40–0.79)	0.91 ± 0.27*	0.86 ± 0.38*	0.85 ± 0.31*
Chromium (μg/L)	< 1.5	0.82 ± 0.17	0.91 ± 0.17	0.91 ± 0.15	1.08 ± 0.30*
Manganese (μg/L)	< 2	0.01 (0.01–0.03)	0.01 (0.01–0.04)	0.01 (0.01–0.03)	0.01 (0.01–0.04)

IFG: Impairied Fasting Glucose, IGT:Impatiring Glucose Tolerance, NIDDM: Noninsulin Dependent Diabetes Mellitus. The results shown as mean ± standart deviation. These results shown as medians (Quarters),

* *P* < 0.05, ND: Nondetectable

**Table 3:** Correlation between HbA1c and trace element levels

		Al	Vn	Cr	Mn	Fe	Ni	Co	Zn	Cu	As	Se	Ag	Cd	Ba	Hg	Pb
Blood	r	0.421	0.312	0.459	0.112	-0.016	0.332	0.160	0.017	0.290	0.032	0.063	0.173	0.161	-0.068	-0.054	0.280
glucose																	
	**P**	<0,001	0.005	<0.001	0.320	0.889	0.002	0.154	0.877	0.008	0.775	0.578	0.122	0.150	0.544	0.628	0.011
HbA1c	r	0.464	0.378	0.538	0.135	0.078	0.401	0.208	0.223	0.418	0.084	0.039	0.244	0.276	0.042	-0.218	0.343
	**P**	<0.001	0.001	<0.001	0.260	0.515	<0.001	0.080	0.060	<0.001	0.485	0.745	0.039	0.019	0.725	0.064	0.003

## Discussion

There has been accumulating evidence that the metabolism of several trace elements is altered in diabetes mellitus and that these nutrients might have specific roles in the pathogenesis and progression of this disease. Metabolic complications appear to be associated with alterations in the levels of some minerals.[1–7] Diabetics have an increased risk of developing renal insufficiency as well as congestive heart failure, independent of hypertensive and coronary atherosclerotic disease.

Diabetes mellitus is the most common cause of end-stage renal disease, accounting for about 40% of cases in many countries. The relationship between long-term environmental Pb exposure and progressive renal insufficiency in patients with type-2 diabetes and diabetic nephropathy is still obscure. Nevertheless, plasma Pb levels were determined to be higher only in NIDDM patients when compared with those in controls. No difference was recorded in the plasma cadmium levels between each group. Several epidemiological studies have demonstrated a positive association between blood Pb levels and age-related decreases in renal function in the general population. This suggests that environmental exposure of low levels of Pb may accelerate the progression of renal dysfunction in healthy people.[8] Bone Pb accumulation may reflect the risk level for impaired renal function, either by serving as a dosimeter of cumulative exposure of the kidney to Pb, or as an indication of the major endogenous source of blood Pb which, in turn, may affect the kidneys. The increase in bone resorption is one of the characteristics of aging in both genders, and aging-associated release of bone Pb into the circulation is a potentially important source of soft-tissue Pb exposure and toxicity. Another factor associated with aging that may increase the nephrotoxicity of Pb is diabetes. The more prevalent form, type-2 diabetes, affects approximately ≥ 10% of the general population (with substantially higher rates at ≥ 55 years of age) and is well known as an independent predictor of accelerated decline in kidney function.[9,10]

In this study, plasma Al levels of the patients with IFG, IGT, and NIDDM were found to be elevated in comparison with the control group. In addition, plasma Al levels were highly correlated with plasma glucose and HbA1c levels [Table 3]. Levine *et al*. in 1990 showed that Al toxicity was commonly found in patients with reduced renal function and that Al had accumulated to a greater level in the tissues of diabetic patients. High Al levels in the context of diabetes mellitus is also associated with decreased myocardial calmodulin activity that may contribute to reduced sarcoplasmic reticulum (Ca + Mg)-ATPase and calcium transport activities. Thus, Al toxicity potentiates the adverse effects of diabetes via a decrease in the sarcoplasmic reticulum calcium uptake.[10]

According to some cross-sectional population studies, diabetes could augment the risk of Cd-induced renal damage, particularly, tubular dysfunction.[11] This was consistent with a previous study that reported that diabetic patients might be more susceptible to the toxic effects of Cd on the renal proximal tubules.[12] Several experimental studies have demonstrated an increased susceptibility towards Cd nephrotoxicity in spontaneously diabetic mice and hamsters when compared with normal animals of the same strain. However, streptozotocin-induced diabetic rats were found to be more susceptible to Cd nephrotoxicity than normal rats when they were subchronically exposed to cadmium chloride in drinking water.[13]

Some authors including Lai *et al.* and Rahman *et al.*, reported the correlation between long-term As exposure and severity of diabetes mellitus.[14,15] However, although our findings revealed higher As levels in patients with DM, IGT, and IFG, no statistically significant correlation was detected between the As levels and the severity of the glucose metabolism disorder.

In 2008, Afridi *et al*, showed that the Pb, Cd, and As levels were significantly higher in scalp hair samples from smoker and nonsmoker diabetic patients than from control subjects.[15] Although the concentrations of these toxic elements were also high in the blood and urine samples of NIDDM patients, the difference was more significant in smoker NIDDM patients.[16]

Zheng *et al*. found that diabetes was found to be strongly associated with Zn deficiency and probably also with Cu deficiency.[17] Hence, zinc supplementation may provide a significant protection against diabetes-induced complications for diabetic individuals. Excessive Fe intake was found to give rise to increased risk of insulin resistance and diabetes whereas excess amounts of Cu and Fe were also found to significantly increase the incidence of diabetic complications. Free radical production is considered as one of the major mechanisms responsible for the toxicity of Cu and Fe. Superoxide radicals generated in all aerobic cells have been attributed to the Fe- and Cu-dependent formation of reactive oxygen species (ROS) through Fenton's reaction.[18] These ROS including hydroperoxyl radicals, singlet oxygen, and hydroxyl radicals are highly reactive and can cause damage to biomolecules. On the other hand, low concentrations of these ROS are required as they act as second messengers, gene regulators, and are even involved in insulin signaling.[19]

Although no difference was noticed between the groups in the plasma levels of iron and zinc, plasma copper levels were found elevated in patients with NIDDM when compared with the controls. A strong correlation between plasma copper and HbA1c levels may indicate an underlying analogy between diabetic complications and this element.

In 2008, Kazi *et al*. reported that Zn, Mn, and Cr levels were significantly reduced in blood and scalp hair samples of diabetic patients; the urinary levels of these elements were found to be higher in diabetic patients. In contrast, high mean values of Cu and Fe concentrations were detected in scalp hair and blood from patients *vs* nondiabetic subjects; however, the differences between the blood sample levels were not significant.[6] In another study, increased Cu levels and decreased Cr levels were observed in diabetic patients.[3] A decrease in zinc and selenium concentrations and an increase in copper concentrations were reported as potentially contributing factors for atherogenicity progression.[4] However, in another study comparing the difference between various blood components in diabetic patients and a control group, Cr was found to be significantly increased in plasma and polymorphonuclear cells. In addition, selenium concentrations were shown to be decreased in the red blood cells of IDDM patients.[5]

Although the routes of action of V, Mn, and Cr have not been thoroughly demonstrated in terms of the pathology of NIDMM, these elements are seldom used for replacement purposes despite some reports favoring their use. Moreover, some research has pointed out the harmful effects of these elements, particularly Cr, on the human metabolism.[5] Chromium (Cr) exists in our environment in several oxidation states, principally as metallic, trivalent, and hexavalent Cr. The highly toxic hexavalent chromium is largely synthesized by the oxidation of the more common and naturally occurring trivalent Cr. Trivalent Cr found in most foods and nutrient supplements, is an essential nutrient that is required in trace quantities for carbohydrate metabolism.

Manganese (Mn) deficiency may result in glucose intolerance similar to diabetes mellitus in some animal species, although studies examining the Mn status of diabetic humans have generated contradictory results. Whole blood Mn levels did not differ significantly between 65 diabetics and 22 nondiabetic controls. Manganese plays a role as a cofactor for the antioxidant enzyme, MnSOD, whose levels are reported to be lower in the white blood cells of diabetics than in those of nondiabetic controls.[20] Although Mn appears to play a role in glucose metabolism, there is little evidence that manganese supplementation improves glucose tolerance in diabetic or nondiabetic individuals.[21] In this regard, we were not able to demonstrate a statistically significant difference between the four groups of this study.

Vanadium (V) is used to strengthen steel in industry and is considered to have no use in humans. However, it has been found to play a role in the treatment of diabetes. Subsequent studies have shown that vanadyl sulfate might be used to lower the elevated blood glucose, cholesterol, and triglyceride levels in a variety of diabetic models including the diabetic rat. Long-term studies of up to one year did not show any toxicity in control or rats administered vanadyl sulfate in doses that lowered elevated blood glucose.[22] In accordance with the previous reports, plasma V levels of the patients with IFG, IGT, and NIDDM in our study were found to be elevated in comparison with the control group. Furthermore, a strong correlated was established between plasma V levels and HbA1c levels.

In the present study, plasma Al, Cr, Ni, V, and Pb levels were found to be increased in the plasma samples from NIDDM patients. Only plasma Al and V levels were significantly higher in patients with IFG and IGT than in the control group. Any correlation between HbA1c levels and toxic elements (especially Cu, Al, V, Ni, Cd, and Pb) may be of great importance to unravel the underlying mechanisms of diabetic complications, although none of these toxic elements reaches the toxic limit.

In summary, this study primarily demonstrates that trace and toxic element levels in plasma of patients with not only NIDDM, but also with IFG and IGT could significantly deviate from control ranges. Therefore, it seems reasonable to analyze their plasma levels in clinically routine practices to monitor the trace element status in patients with NIDDM and other related disorders.

These results obtained are consistent with those obtained in other studies confirming that metals may play significant roles in the development of diabetes mellitus and progression of its metabolic complications. Nevertheless, comprehensive studies covering larger populations are needed to elucidate a clear relationship between glucose metabolism disorders and plasma levels of trace metal levels.
